# Impact of male factors on morphokinetic parameters: a prospective analysis using time-lapse monitored embryos

**DOI:** 10.1007/s10815-025-03658-4

**Published:** 2025-09-17

**Authors:** Rossella Cannarella, Claudia Leanza, Sandrine Chamayou, Andrea Crafa, Federica Barbagallo, Antonino Guglielmino, Sandro La Vignera, Aldo E. Calogero

**Affiliations:** 1 Department of Clinical and Experimental Medicine, University of Catania, Via S. Sofia 78, 95123 Catania, Italy; 2 Glickman Urological and Kidney Institute, Cleveland Clinic, Cleveland, OH USA; 3 Centro HERA - Unità Di Medicina Della Riproduzione, Catania, Italy

**Keywords:** ART, Assisted reproductive technology, Male, Paternal BMI, Body mass index, Male age, Paternal age, Sperm parameters

## Abstract

**Introduction:**

Time-lapse technology enables recording embryo morphokinetic parameters, which are associated with embryonic competence and assisted reproductive technology (ART) outcomes. While female factors such as age and BMI are known to influence these parameters, the role of male factors remains understudied.

**Aim:**

This study aimed to evaluate the influence of male factors on preimplantation embryo morphokinetics.

**Methods:**

In this prospective observational study, 1,210 embryos from infertile couples undergoing Intracytoplasmic sperm injection (ICSI) or intracytoplasmic morphologically-selected sperm injection (IMSI) were monitored using time-lapse imaging. Male data, including age, BMI, sperm concentration, and sperm DNA fragmentation (SDF) were collected. Multiple regression analysis assessed the association between paternal factors and morphokinetic parameters, adjusting for female confounders.

**Results:**

After adjustment, male age and BMI were found to significantly influence embryo developmental stages (from time to pronuclei appearance to t4 and t6 for age, from time to pronuclei appearance to t2 and t8 for BMI). The impact of sperm concentration was less consistent, and no significant relationship was observed with SDF.

**Conclusions:**

These findings highlight the role of male factors, particularly age and BMI, in influencing embryo morphokinetics, even after accounting for female confounders. This underscores the potential for clinical interventions targeting paternal health to optimize ART outcomes. Additionally, the study reinforces the importance of considering both parental contributions in ART success, particularly the increasingly recognized influence of male age.

**Supplementary Information:**

The online version contains supplementary material available at 10.1007/s10815-025-03658-4.

## Introduction

In assisted reproductive technologies (ART), both static and dynamic morphological assessments of oocytes and embryos are essential for selecting those with the highest implantation potential [1]. Oocyte evaluation focuses on features such as the cumulus-oocyte complex, zona pellucida, perivitelline space, polar body, vacuolization, refractile bodies, and overall size, granularity, shape, and color to predict the developmental competence [1]. According to the Istanbul Consensus, an ideal fertilized oocyte is spherical with two polar bodies and two centrally located, equally sized pronuclei [1]. Similarly, embryo grading assesses viability and implantation potential based on criteria such as blastomere number, size and shape,fragmentation,cell cleavage and the morphology of the inner cell mass and trophectoderm. However, despite these detailed assessments, the correlation between morphological features and actual embryonic competence remains uncertain due to significant intra- and inter-observer variability [2].

To improve embryo selection, a range of non-invasive and invasive techniques are used to select embryos. These include preimplantation genetic testing, proteomics, metabolomics, oxygen consumption, and measurement of oxidative stress in the culture medium [3]. Moreover, in recent decades, there has been increasing recognition of the importance of linking embryo morphology with their kinetics, leading to the development of a method known as embryo morphokinetics [1]. Embryos may now be classified and selected for transfer based on their in vitro morphokinetic parameters [4], as early embryo kinetics are thought to predict embryonic competence [5]. Time-lapse technology allows for near-continuous observation of embryo morphokinetics through frequent image acquisition [6]. Various devices are available for recording embryo morphokinetics, although not all capture the full range of embryo parameters.

According to the 2014 guidelines [6], key morphokinetic markers include time of insemination (t0), pronuclear appearance (tPNa), fading (tPNf), first cleavage (t2), subsequent divisions (t3–tn), morula formation (tM), blastocyst formation (tB), and expansion (tEB). Abnormal events such as reverse or direct cleavage may also be observed [6].

Several studies have Linked embryo kinetics to clinical outcomes. A large retrospective study of 9,450 ICSI cycles found that delayed blastocyst development correlated with higher cleavage anomalies and reduced implantation, pregnancy, and live birth rates, even after adjusting for female age [5]. Embryo development is influenced by gene expression and culture conditions [7, 8], as well as maternal factors such as age and BMI. For example, oocytes from younger women reach key developmental milestones more quickly, and women who conceived had embryos with faster morphokinetics, regardless of age [5, 9]. Overweight and obesity have been linked to slower embryo development and delayed cleavage times [10].

In contrast, few studies have investigated the role of paternal factors in embryo morphokinetics. One study involving 429 fertilized oocytes found that lower sperm concentration was associated with delayed tPB2, suggesting an effect of oligozoospermia on early embryo development [11]. Based on these findings, the present study aims to evaluate the impact of male factors—including age, body mass index (BMI), sperm concentration, and sperm DNA fragmentation (SDF)—on early and late morphokinetic parameters and ART outcomes.

## Patients and methods

### Study protocol

This prospective observational study involved infertile couples referred to an ART center. Data collected from female partners included infertility etiology, age, BMI, serum levels of luteinizing hormone (LH), follicle-stimulating hormone (FSH), 17β-estradiol (E_2_), progesterone, and anti-Müllerian hormone (AMH). For male partners, data collected included age, BMI, sperm concetration and SDF.

Prospective data collection encompassed sperm concentration, type of ART used [ICSI, or intracytoplasmic morphologically-selected sperm injection (IMSI)], type of controlled ovarian hyperstimulation (COH) protocol used, oocyte morphology and quality, embryo kinetics, embryo morphology, day of embryo transfer (ET), serum β-human chorionic gonadotropin (βhCG) levels post-ET, pregnancy type and timing (biochemical, extrauterine, or clinical), miscarriage rates, and live birth outcomes.

### Patient selection

Inclusion criteria for couples were:

- Use of homologous ART (i.e., using the gametes of both partners in the couple)
- Infertility duration of ≥ 12 months
- Male idiopathic infertility or female infertility due to anovulation or tubal factor

Exclusion criteria for couples were:

- Female infertility due to reduced ovarian reserve (e.g., severe endometriosis or premature ovarian failure)
- Repeated implantation failure
- Recurrent pregnancy loss
- Use of heterologous ART (i.e., involving donor sperm or oocytes)

Additionally, individual eligibility criteria were applied to each partner, as summarized in Supplementary Table 1.

### Oocyte collection

COH was induced using either long or short agonist protocol. Specifically, gonadotropin-releasing hormone analog (buserelin, Suprefact®, Hoechst Marion Roussel Deutschland GmbH, Frankfurt, Germany) was administered during the luteal phase of the preceeding cycle, followed by recombinant FSH (Gonal-F®, Merck-Serono, London, UK, or Puregon®, MSD, Franklin Lakes, USA) starting on day 3 of the current cycle. Thirty-five hours after the administration of 10,000 IU hCG (Gonasi®, IBSA, Italy), ultrasound-guided transvaginal aspiration of the oocyte-cumulus complexes was performed.

### Sperm collection

After 3–4 days of sexual abstinence, male partners collected semen samples in sterile containers via masturbation and allowed to liquefy. Conventional sperm parameters were evaluated according to the most recent World Health Organization criteria (WHO, 2021). Following liquefaction, spermatozoa were isolated using density gradient centrifugation, according to the manufacturer’s instructions (SpermGardTM, Vitrolife, Englewood, CO, USA) [12]. Motile spermatozoa were selected for ART. All semen samples were processed uniformly using the same protocols and reagents to ensure consistency across the study.

### Embryo kinetics

T0 corresponds to the moment when a spermatozoon is injected into the oocyte. tPNa is the time at which of both PN appear, and tPNf marks the time of the last observation of the two PN. t2, t3, t4, …, tn represent the times at which embryo raches stages 2, 3, 4, …, n-cell division. tM denotes the time when the embryo reaches the morula stage. tB corresponds to time in which a crescent-shaped area begins to emerge from the morula. tEB is the time in which the embryo reaches the expanded blastocyst stage, characterized by an increase in volume and visibile expansion of the blastocoel cavityis. For data analysis, each of these times was expressed in hours and fractions of an hour [6] (Supplementary Fig. 1). All annotations were performed by trained embryologists according to standardized protocols. Regular inter-observer calibration sessions were held to ensure consistency and minimize discrepancies. Ambiguous or borderline cases were reviewed jointly to reach consensus decisions, thus enhancing the reliability of the timing assessments.

### Study end-points

Male age, BMI, sperm concentration, and SDF rate were collected and analyzed for their correlation with embryo kinetic parameters (tPN to tEB).

### Statistical analysis

Data are shown as mean ± standard deviation (SD) for normally distributed variables, and as median with interquartile range (IQR) for non-normally distributed continuous variables throughout the manuscript. Data distribution was assessed with the Shapiro-Wilks test. The relationship between primary outcomes and embryo morphokinetics was evaluated using two stepwise multiple regression models. The first model included male age, BMI, and sperm concentration, along with female age, BMI, and AMH as independent variables, with morphokinetic parameters as the dependent variables. To avoid overfitting, the second model, designed to address for the limited data on SDF, included male age, BMI, SDF, female age, BMI, and AMH as independent variables, with morphokinetic parameters as the dependent variables. Statistical analysis was performed using MedCalc Software Ltd. (Ostend, Belgium), version 19.6–64-bit. A *p*-value of less than 0.05 was considered statistically significant.

### Ethical approval

The study was conducted at the ###, and at the ART center ###. The study protocol was approved by the Ethics Committee ### (approval number 18189, approved on March 27, 2023). Informed consent was obtained from alla participants, who were fully informed about the purpose of the study purpose and the nature of the procedures involved. The study was conducted according to the principles ourlined in the Declaration of Helsinki.

## Results

### General results

Data of 151 participants undergoing ICSI or IMSI were analyzed, including a total of 1,210 embryos. Infertility was ascribed to a male factor in 71 cases (47.0%), anovulation in 19 cases (12.6%), tubal factor in 41 cases (27.2%), and idiopathic in 20 cases (13.2%). The mean age and BMI of male partners were 38.1 ± 5.8 years and 26.5 ± 3.3 kg/m^2^, respectively. Female partners had a mean age of 35.6 ± 4.7 years, a mean BMI of 23.6 ± 4.2 kg/m^2^, and mean serum AMH levels of 2.97 ± 2.03 ng/mL. Descriptive characteristics of the enrolled cohort are shown in Table 1.

**Table 1:** Descriptive characteristics of the enrolled cohort

Parameter	Mean ± SD or %	Min–Max
Male partners		
Age (years)	38.1 ± 5.8	24–61
BMI (Kg/m^2^)	26.5 ± 3.3	17.9–38.7
Obesity (BMI ≥ 30 kg/m^2^)	20.1%	-
Overweight (25 kg/m^2^ < BMI < 30 kg/m^2^)	47.9%	-
Normal weight (18 kg/m^2^ ≤ BMI < 30 kg/m^2^)	32.0%	-
Sperm concentration (mil/mL)	21.5 ± 32.4	0.3–190.0
Oligozoospermia	62.1%	
SDF (%)	19.0 ± 12.0	5.0–58.0
Post-gradient sperm concentration (mil/mL)	3.9 ± 6.6	0.2–40.0
Female partners		
Age (years)	35.6 ± 4.7	21.4–46.5
Age ≤ 35 years	48.3%	-
35 years < Age ≤ 40 years	31.1%	-
Age > 40 years	20.6%	-
BMI (Kg/m^2^)	23.6 ± 4.2	16.8—39.8
Obesity (BMI ≥ 30 kg/m^2^)	5.8%	-
Overweight (25 kg/m^2^ < BMI < 30 kg/m^2^)	27.6%	-
Normal weight (18 kg/m^2^ ≤ BMI < 30 kg/m^2^)	66.6%	-
AMH (ng/mL)	2.97 ± 2.03	0.9–9.0
ART characteristics		
Short acting protocol	49.0%	-
Long acting protocol	51.0%	-
IMSI	8.4%	-
ICSI	91.6%	-
n. of aspirated oocytes (per cycle)	11.5 ± 3.4	4—19
Morphokinetic parameters		
tPNa	7.7 ± 1.7	4.6–16.9
tPNf	25.5 ± 4.1	16.8–43.9
t2	28.4 ± 4.7	18.6–46.2
t3	38.1 ± 5.4	20.8–60.8
t4	39.9 ± 5.8	24.7–69.8
t5	51.8 ± 7.9	30.5–83.1
t6	54.3 ± 7.7	30.9–87.5
t7	57.7 ± 8.3	38.4–91.3
t8	60.5 ± 9.2	39.0–91.3
t9	71.7 ± 9.5	47.4–100.2
tM	84.8 ± 9.6	57.3–117.6
tB	99.2 ± 8.0	76.9–118.5
tEB	106.4 ± 6.6	84.9–118.7

*AMH* anti-Müllerian hormone, *BMI* body mass index, *ICSI* intracytosplasmic sperm injection, *IMSI* intracytoplasmic morphologically-selected sperm injection, *SD* standard deviation, *SDF* sperm DNA fragmentation, *tPNa* time of pronuclei appearance, *tPNf* time of pronuclei fading, *tM* Time of morula, *tB* When the frame showed a crescent-shaped area began to emerge from the morula, *tEB* Time when an increase in volume and expansion of the blastocoel cavity was visible

### Multiple regression analysis

After adjusting for both female (age, BMI, and AMH) and male (BMI and sperm concentration) factors, male age was significantly associated with earlier embryo morphokinetics timings (tPNa, tPNf, t2, t3, t4, t6) (Table 2). These findings suggest that advanced male age may be Linked to delayed embryo development, particularly up to the 6-cell stage.

**Table 2:** Multiple regression analysis of the association between paternal factors and morphokinetic parameters

	Adjusted model					
		Coefficient	Standard error	*p*-value	r_partial_	r_semipartial_
tPNa (*n* = 362)	Variable	4.9421				
	Male age	0.093	0.039	**0.0177**	0.199	0.194
	Male BMI	0.086	0.035	**0.014**	0.201	0.200
	Sperm concentration	−0.000	0.000	**0.040**	−0.173	0.168
	Female age	−0.039	0.044	0.383	−0.074	0.071
	Female BMI	0.033	0.048	0.494	0.058	0.055
	Female AMH	−0.020	0.062	0.751	−0.027	0.026
tPNf (*n* = 907)	Variable	28.1709				
	Male age	0.192	0.052	**0.000**	0.191	0.185
	Male BMI	−0.181	0.062	**0.004**	−0.151	0.146
	Sperm concentration	6.214	6.669	0.352	0.049	0.047
	Female age	−0.087	0.065	0.177	−0.071	0.068
	Female BMI	−0.155	0.061	**0.011**	−0.133	0.128
	Female AMH	0.092	0.098	0.349	0.049	0.047
t2 (*n* = 893)	Variable	30.0655				
	Male age	0.247	0.069	**0.000**	0.184	0.180
	Male BMI	−0.222	0.081	**0.006**	−0.143	0.138
	Sperm concentration	0.000	0.000	0.980	0.001	0.001
	Female age	−0.081	0.087	0.351	−0.049	0.047
	Female BMI	−0.137	0.079	0.086	−0.089	0.086
	Female AMH	0.053	0.126	0.678	0.022	0.021
t3 (*n* = 837)	Variable	37.6224				
	Male age	0.157	0.073	**0.033**	0.116	0.115
	Male BMI	−0.068	0.084	0.420	−0.044	0.043
	Sperm concentration	0.000	0.000	0.297	0.057	0.056
	Female age	−0.080	0.093	0.388	−0.047	0.046
	Female BMI	−0.116	0.084	0.166	−0.075	0.074
	Female AMH	0.127	0.134	0.345	0.051	0.050
t4 (*n* = 790)	Variable	37.9650				
	Male age	0.207	0.083	**0.014**	0.137	0.135
	Male BMI	−0.103	0.096	0.283	−0.059	0.059
	Sperm concentration	0.000	0.000	0.581	0.031	0.030
	Female age	−0.077	0.105	0.464	−0.041	0.040
	Female BMI	−0.091	0.094	0.0335	−0.054	0.053
	Female AMH	0.117	0.149	0.435	0.044	0.043
t5 (*n* = 670)	Variable	47.2015				
	Male age	0.208	0.118	0.079	0.108	0.108
	Male BMI	−0.003	0.131	0.980	−0.002	0.002
	Sperm concentration	0.000	0.000	0.733	0.021	0.021
	Female age	−0.126	0.146	0.390	−0.053	0.053
	Female BMI	−0.073	0.133	0.581	−0.034	0.033
	Female AMH	0.247	0.216	0.254	0.070	0.070
t6 (*n* = 644)	Variable	50.2433				
	Male age	0.224	0.113	**0.049**	0.123	0.122
	Male BMI	0.059	0.126	0.637	0.030	0.029
	Sperm concentration	0.000	0.000	0.598	0.033	0.033
	Female age	−0.148	0.140	0.293	−0.066	0.065
	Female BMI	−0.145	0.127	0.257	−0.071	0.070
	Female AMH	0.071	0.211	0.737	0.021	0.021
t7 (*n* = 603)	Variable	47.4811				
	Male age	0.220	0.138	0.113	0.102	0.100
	Male BMI	0.248	0.154	0.110	0.103	0.101
	Sperm concentration	0.000	0.000	0.653	0.029	0.029
	Female age	−0.176	0.171	0.305	−0.066	0.065
	Female BMI	−0.068	0.156	0.663	−0.028	0.028
	Female AMH	0.215	0.256	0.402	0.054	0.053
t8 (*n* = 587)	Variable	49.4432				
	Male age	0.309	0.157	0.051	0.127	0.123
	Male BMI	0.408	0.175	**0.020**	0.151	0.147
	Sperm concentration	0.000	0.000	0.103	0.106	0.103
	Female age	−0.325	0.193	0.093	−0.109	0.106
	Female BMI	−0.137	0.177	0.439	−0.050	0.049
	Female AMH	0.128	0.291	0.661	0.029	0.028
t9 (*n* = 533)	Variable	62.9419				
	Male age	0.052	0.181	0.773	0.020	0.020
	Male BMI	0.233	0.205	0.256	0.078	0.078
	Sperm concentration	0.000	0.000	0.728	0.024	0.024
	Female age	−0.090	0.219	0.682	−0.028	0.028
	Female BMI	0.068	0.209	0.747	0.022	0.022
	Female AMH	0.086	0.325	0.790	0.018	0.018
tM (*n* = 508)	Variable	82.8815				
	Male age	0.259	0.177	0.145	0.103	0.100
	Male BMI	0.049	0.203	0.810	0.017	0.016
	Sperm concentration	0.000	0.000	0.725	0.025	0.024
	Female age	−0.128	0.216	0.553	−0.042	0.041
	Female BMI	−0.174	0.198	0.380	−0.062	0.060
	Female AMH	−0.843	0.323	**0.010**	−0.182	0.178
tB (*n* = 414)	Variable	91.9780				
	Male age	0.053	0.157	0.736	0.026	0.025
	Male BMI	0.167	0.175	0.342	0.074	0.071
	Sperm concentration	0.000	0.000	0.114	0.123	0.119
	Female age	0.098	0.189	0.606	0.040	0.039
	Female BMI	−0.189	0.174	0.280	−0.084	0.081
	Female AMH	−0.288	0.283	0.311	−0.079	0.076
tEB (*n* = 274)	Variable	106.6724				
	Male age	−0.120	0.150	0.425	−0.072	0.067
	Male BMI	−0.064	0.185	0.730	−0.031	0.029
	Sperm concentration	−0.000	0.000	0.570	−0.052	0.048
	Female age	0.363	0.186	0.054	0.174	0.163
	Female BMI	−0.246	0.160	0.126	−0.138	0.129
	Female AMH	−0.670	0.260	**0.011**	−0.228	0.216

*AMH* Anti-Müllerian hormone, *BMI* Body mass index, *tPNa* time of pronuclei appearance, *tPNf* time of pronuclei fading, *tM* Time of morula, *tB* When the frame showed a crescent-shaped area began to emerge from the morula, *tEB* Time when an increase in volume and expansion of the blastocoel cavity was visible

Male BMI was also associated with variations in embryo morphokinetics, specifically tPNa, tPNf, t2, and t8 (Table 2). In addition, sperm concetration was associated with tPNa (Table 2).

In a separate model adjusted only for female variables (age, BMI, and AMH), applied to the subgroup with available SDF data, no significant associations were found between SDF and any embryo morphokinetic parameter (Supplementary Table 2).

## Discussion

The present study aimed to investigate the potential influence of male parameters (age, BMI, sperm concentration, and SDF) on embryo morphokinetic parameters, which are associated with the likelihood of reaching the blastocyst stage. After adjusting for female confounders using multiple regression analysis, male age and BMI were found to influence embryo developmental stages (from tPNa to t4 and t6 for age, from tPNa to t2 and t8 for BMI). The effect of sperm concentration was less consistent, showing significant associations only with tPNa, while no significant relationship was observed with the SDF rate. These findings contribute to a better understanding of the male influence on embryo morphokinetics.

Since the advent of time-lapse monitoring, studies have linked early morphokinetic parameters with blastocyst formation and implantation potential Motato et al. [13] found that embryos reaching the blastocyst stage cleaved earlier than those that did not. Meseguer et al. [14] showed improved pregnancy rates with time-lapse–guided embryo selection. Basile et al. [15] identified t3, cc2, and t5 as key predictors of implantation and developed an embryo grading algorithm accordingly. Early morphokinetics may thus influence both blastocyst development and ART outcomes.

Evidence from animal studies has demonstrated that environmental factors can modify sperm epigenetics, thereby influcencing not only embryo development but also offspring health. For example, obesity has been shown to alter germline DNA methylation profiles in mice [16], affect fetal and placental gene expression [17], and modulate offspring hepatic gluconeogenesis via *IGF2*/*H19* gene methylation changes [18]. Similar findings have been reported for male age [19–21]. In addition to DNA methylation and histone modifications, RNAs represent another key component of epigenetic regulation. Sperm-derived RNAs, such as IGF2 mRNA, have recently been implicated in modulating early embryo morphokinetics, particularly during the t2-t5 stages [22], for review see: [23]. Other studies have shown that small RNAs acquired during epididymal transit are essential for normal preimplantation embryo development [24]. Interestingly, exposure to elevated temperatures has been found to modify the sperm epigenome, resulting in accelerated early embryo development in mice [25]. This growing body of evidence provides a complelling rationale for hypothesizing a role for male factors in influencing embryo morphokinetics.

Male age has increased in industrialized countries due to various socio-economic and cultural factors. Advanced male age has been associated with impaired sperm quality, as well as poorer outcomes in ART and offspring health [26–28]. However, the impact of male age on pregnancy outcomes remains less understood. In this context, our study aimed to evaluate the influence of male age on embryo morphokinetic parameters, which may serve as a potential mechanism underlying ART outcomes. To the best of our knowledge, only one other prior study has investigated the effect of male age on embryo morphokinetics, showing its direct association with delayed cell cleavage and blastulation, as well as an indirect effect on clinical pregnancy and live-birth rates [29, 30]. In a recent cohort study (2017–2023), analyzing over 47,000 metaphase II oocytes from 5,847 IVF cycles, increasing paternal age showed a negative, independent association with euploidy, particularly in men over 30 years of age, even after adjusting for sperm origin and quality parameters [31]. Furthermore, the study highlighted how low sperm concentration (< 1 million/mL), use of testicular sperm, poor motility (≤ 25%), and the use of frozen sperm samples associate with reduced fertilization and lower euploidy rates per biopsied blastocyst [31]. These findings highlight the influence of paternal variables not only on the initiation of embryonic development but also on the accurate progression through morphokinetic milestones leading to a euploid blastocyst.

Overweight and obesity, driven by a combination of genetic, dietary, and lifestyle factors, have become widespread global issues with significant health implications, including adverse effects on reproduction [32], Ng et al., 2013). Currently, more than half of men of reproductive age are either overweight or obese [33]. The impact of obesity on female reproductive health has been extensively studied, showing its detrimental influence on oocyte and embryo quality [34], as well as on female and fetal health [35]. Similarly, research on male reproductive health has highlighted the negative influence of obesity on sperm quality and male fertility [22]. Additionally, male obesity has been shown to affect ART outcomes and neonatal health. Hoek and colleagues observed that higher male BMI was associated with accelerated preimplantation embryo development, particularly during the early cleavage divisions. However, the authors also reported an inverse association between male BMI and fertilization rate, though no effect was seen on live birth rate [36]. In line with these findings, our data showed a significant inverse correlation between male BMI and tPNa, tPNf, t2, and t8. In patients with oligozoospermia or asthenozoospermia, male BMI was negatively correlated with transferable and high-quality embryos on day 3 [37]. Moreover, male obesity has been associated with poorer IVF outcomes (Yang et al., 2016), and higher male preconception BMI has been associated with an increased rate of macrosomia and large-for-gestational age offspring [37, 38].

The mechanisms through which excess body weight impacts embryo morphokinetics remain unclear. Evidence from animal studies suggests that obesity may induce epigenetic alterations in sperm at loci relevant to embryo development, such as *Wnt*, *TGF-ß*, and *Notch* (Deshpande et al., 2020). A recent review has further emphasized the role of epigenetic regulation in early human embryo development, highlighting the mechanisms underlying developmental plasticity [39].

Regarding SDF rate, our findings differ from previous reports. Wang and colleagues found that an SDF ≥ 15% negatively affected certain morphokinetic parameters and reduced fertilization rate in ICSI cycles [40]. Similarly, Wdowiak et al. [41] reported that lower SDF rate were associated with faster progression to blastocyst and higher pregnancy rates. Setti et al. [30] observed slower development in embryos derived from sperm samples with SDF > 30%, while Esbert et al. [42] reported delayed cleavage in embryos from donated oocytes but not from autologous oocytes in the highest SDF quartile. In contrast, Anbari et al. [43] found no negative impact of high SDF on embryo morphokinetics in conventional IVF cycles. Our study differs from these prior studies in that SDF analysis was not performed in all patients and was not the primary outcome. Thus, results might differ in a larger, specifically designed cohort. Importantly, earlier studies did not adjust for female confounders such as age, BMI, and AMH, which we included in our analysis. In our multiple regression analysis, no significant correlation was found between SDF and morphokinetic parameters. While no significant relationship emerged after adjustment, a potential influence of high SDF on embryo development cannot be excluded. Factors such as obesity [18, 44] or advanced male age [45] may indeed influence other molecular aspects of sperm quality—particularly epigenetic modifications such as DNA methylation patterns and sperm RNA content. These epigenetic marks can be transmitted to the embryo at fertilization and may affect early developmental processes and implantation potential [22, 23, 46]. Thus, even in the presence of normal standard sperm parameters, male characteristics may still play a critical role in shaping embryonic competence through non-genetic mechanisms.

Our findings, if confirmed by further research, could expand the understanding of how preconceptional male age and BMI influence embryo morphokinetics, providing a foudation for early clinical intervention before couples undergo ART. However, it is important to note that the design of our study does not allow us to infer causalty between male factors and outcomes. Nevertheless, adjusting for confounding factors, particularly female age, BMI, and AMH, strengthens the reliability of our findings and underscore the need for additional prospective studies.

A potential limitation of the study is that different controlled ovarian stimulation protocols were used among participants, which were not included in the regression analysis. Additionally, we did not adjust for oocyte quality due to data unavailability, which may have introduced bias. The inherent subjectivity in annotating specific morphokinetic parameters, particularly tPNa and tEB, is another shortcoming. However, to minimize inter-observer variability, all annotations were performed by trained embryologists following standardized procedures, and regular calibration sessions were held to ensure consistency and accuracy. Finally, the use of sperm injection as the reference point for time zero (t0) may affect comparability with other studies using alternative definitions, as acknowledged by ESHRE guidelines (Apter et al., 2020).

Among the key strengths of our study is the comprehensive multivariate analysis that accounted for relevant female confounders, including age, BMI, and AMH—an approach often overlooked in previous studies on this topic. Additionally, our dataset includes precise and standardized morphokinetic annotations performed by experienced embryologists using a validated time-lapse platform. Importantly, our work is among the few available studies to explore the impact of multiple male factors—age, BMI, sperm concentration, and SDF—on embryo morphokinetics within the same analysis, offering a more integrated view of male influence. Furthermore, the relatively large sample size and strict inclusion criteria enhance the generalizability of our findings in a real-world ART setting.

## Conclusions

This study supports the influence of male age and BMI on specific stages of embryo morphokinetics, highlighting the role of male factors in preimplantation embryo development. However, the current level of evidence remains limited. Prospective studies with larger sample sizes—particularly in targeted populations such as oocyte donors—are needed to further validate and clarify these findings. A better understanding of male contributions could inform early clinical interventions aimed at optimizing ART outcomes .

## Supplementary Information

Below is the link to the electronic supplementary material.

Supplementary file1 (PNG 481 KB)

Supplementary file1 (DOCX 49.3 KB)

## Data Availability

Data are available upon request to the corresponding author and will also be made available to the editors of the journal for review or query upon request.
