# Supplementary figures and images for: Impact of male factors on morphokinetic parameters: a prospective analysis using time-lapse monitored embryos
Source: J Assist Reprod Genet. 2025 Sep 17;42(10):3551–60. doi: 10.1007/s10815-025-03658-4 (PMC12602791; doi:10.1007/s10815-025-03658-4)

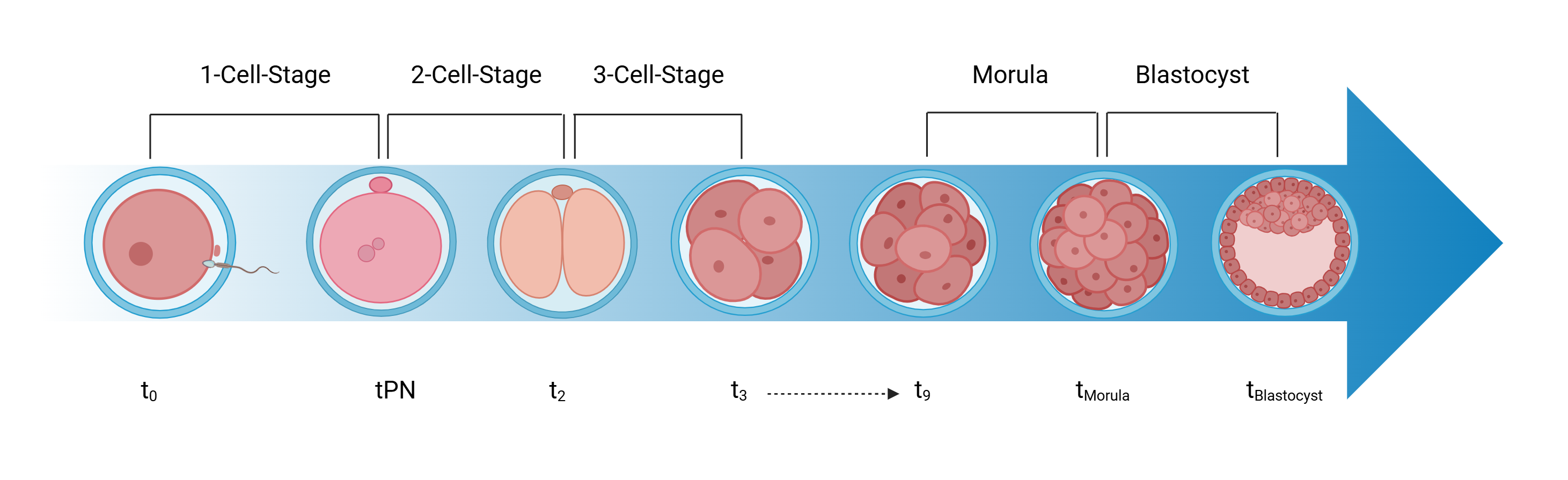

Supplement: Supplementary file 1 — Supplementary file1 (PNG 481 KB) [file 10815_2025_3658_MOESM1_ESM.png]
