# Supplementary material for: Impact of male factors on morphokinetic parameters: a prospective analysis using time-lapse monitored embryos
Source: J Assist Reprod Genet. 2025 Sep 17;42(10):3551–60. doi: 10.1007/s10815-025-03658-4 (PMC12602791; doi:10.1007/s10815-025-03658-4)
Supplement: Supplementary file 2 — Supplementary file1 (DOCX 49.3 KB) [file 10815_2025_3658_MOESM2_ESM.docx]

**Supplementary information**

**Supplementary Table 1.** Selection criteria

| **Criteria** | **Female Partners** | **Male Partners** |
| --- | --- | --- |
| Age | ≥18 years | ≥18 years |
| Major comorbidities/organ failure | None (heart, kidney, liver failure, diabetes, tumors, thyroid diseases, hyperprolactinemia) | None (heart, kidney, liver failure, diabetes, tumors) |
| Lifestyle factors | No current/past alcohol abuse, drug use, cigarette smoking | No current/past alcohol abuse, drug use, cigarette smoking |
| Menstrual cycle | Regular (25–35 days) | N/A |
| Serum AMH level | >0.83 ng/mL | N/A |
| Ovarian response | ≥5 mature follicles at retrieval | N/A |

**Supplementary Table 2.** Multiple regression analysis of the association between paternal factors and morphokinetic parameters.

|  | **Adjusted model** | | | | | |
| --- | --- | --- | --- | --- | --- | --- |
|  |  | **Coefficient** | **Standard error** | **p-value** | **r_partial_** | **r_semipartial_** |
| **tPNa**  **(n = 37)** | **Variable** | 10.3865 |  |  |  |  |
|  | **Male age** | 0.041 | 0.000 | **-** | - | -3.1x10^13^ |
|  | **Male BMI** | -0.418 | 0.000 | 1.0 | 0.000 | 0.000 |
|  | **SDF** | -0.151 | 0.000 | 1.0 | 0.000 | 0.000 |
|  | **Female age** | -0.098 | 0.000 | 1.0 | 0.000 | 0.000 |
|  | **Female BMI** | -0.122 | 0.000 | 1.0 | 0.000 | 0.000 |
|  | **Female AMH** | 1.714 | 0.000 | 1.0 | 0.000 | 0.000 |
| **tPNf**  **(n = 103)** | **Variable** | 44.5555 |  |  |  |  |
|  | **Male age** | 0.376 | 0.368 | 0.315 | 0.190 | 0.149 |
|  | **Male BMI** | -0.965 | 0.595 | 0.116 | -0.293 | 0.236 |
|  | **SDF** | -0.025 | 0.134 | 0.853 | -0.035 | 0.027 |
|  | **Female age** | -0.503 | 0.301 | 0.853 | -0.035 | 0.027 |
|  | **Female BMI** | 0.641 | 0.502 | 0.212 | 0.235 | 0.186 |
|  | **Female AMH** | -1.673 | 0.877 | 0.067 | -0.339 | 0.278 |
| **t2**  **(n = 107)** | **Variable** | 60.5392 |  |  |  |  |
|  | **Male age** | -0.141 | 0.420 | 0.739 | -0.059 | 0.043 |
|  | **Male BMI** | -0.968 | 0.690 | 0.170 | -0.241 | 0.178 |
|  | **SDF** | 0.100 | 0.147 | 0.502 | 0.119 | 0.086 |
|  | **Female age** | -0.468 | 0.359 | 0.202 | -0.225 | 0.165 |
|  | **Female BMI** | 0.997 | 0.590 | 0.101 | 0286 | 0.214 |
|  | **Female AMH** | -3.163 | 0.065 | **0.006** | -0.465 | 0.376 |
| **t3**  **(n = 101)** | **Variable** | 54.8709 |  |  |  |  |
|  | **Male age** | -0.182 | 0.395 | 0.649 | -0.084 | 0.069 |
|  | **Male BMI** | -0.729 | 0.654 | 0.273 | -0.200 | 0.169 |
|  | **SDF** | 0.019 | 0.140 | 0.893 | 0.025 | 0.020 |
|  | **Female age** | 0.049 | 0.333 | 0.884 | 0.027 | 0.022 |
|  | **Female BMI** | -0.415 | 0.552 | 0.459 | 0.136 | 0.113 |
|  | **Female AMH** | -1.377 | 0.982 | 0.171 | -0.248 | 0.212 |
| **t4**  **(n = 99)** | **Variable** | 60.920 |  |  |  |  |
|  | **Male age** | -0.190 | 0.456 | 0.680 | -0.077 | 0.068 |
|  | **Male BMI** | -0.792 | 0.754 | 0.302 | -0.192 | 0.173 |
|  | **SDF** | -0.039 | 0.162 | 0.811 | -0.045 | 0.140 |
|  | **Female age** | -0.091 | 0.384 | 0.814 | -0.044 | 0.039 |
|  | **Female BMI** | -0.547 | 0.637 | 0.397 | 0.158 | 0.141 |
|  | **Female AMH** | -1.553 | 1.135 | 0.182 | -0.246 | 0.225 |
| **t5**  **(n = 89)** | **Variable** | 22.8825 |  |  |  |  |
|  | **Male age** | 2.423 | 0.932 | **0.016** | 0.461 | 0.384 |
|  | **Male BMI** | -2.890 | 1.344 | **0.042** | -0.395 | 0.318 |
|  | **SDF** | 0.219 | 0.278 | 0.438 | 0.156 | 0.117 |
|  | **Female age** | -0.517 | 0.619 | 0.414 | -0.164 | 0.123 |
|  | **Female BMI** | 0.934 | 1.016 | 0.367 | 0.181 | 0.136 |
|  | **Female AMH** | 1.412 | 1.937 | 0.473 | 0.144 | 0.108 |
| **t6**  **(n = 87)** | **Variable** | 35.960 |  |  |  |  |
|  | **Male age** | 2.382 | 0.975 | **0.022** | 0.439 | 0.379 |
|  | **Male BMI** | -2.599 | 1.406 | 0.076 | -0.347 | 0.287 |
|  | **SDF** | 0.035 | 0.290 | 0.905 | 0.024 | 0.019 |
|  | **Female age** | -0.838 | 0.647 | 0.207 | -0.251 | 0.201 |
|  | **Female BMI** | 0.903 | 1.062 | 0.403 | 0.168 | 0.132 |
|  | **Female AMH** | 0.772 | 2.024 | 0.706 | 0.076 | 0.059 |
| **t7**  **(n = 82)** | **Variable** | -19.408 |  |  |  |  |
|  | **Male age** | 1.979 | 0.000 | - | - | -1.1x10^13^ |
|  | **Male BMI** | -0.077 | 0.000 | - | - | -4.3x10^11^ |
|  | **SDF** | 0.275 | 0.000 | - | - | -2.9x10^11^ |
|  | **Female age** | - | - | - | - | - |
|  | **Female BMI** | -0.344 | 0.000 | - | - | -6.3x10^8^ |
|  | **Female AMH** | 2.519 | 0.000 | - | - | -1.5x10^13^ |
| **t8**  **(n = 79)** | **Variable** | -1.9501 |  |  |  |  |
|  | **Male age** | 0.586 | 0.000 | - | - | -1.1x10^13^ |
|  | **Male BMI** | 1.317 | 0.000 | - | - | -1.3x10^13^ |
|  | **SDF** | 0.204 | 0.000 | - | - | -6.6x10^11^ |
|  | **Female age** | 0.593 | 0.000 | - | - | -2.7x10^13^ |
|  | **Female BMI** | -0.813 | 0.000 | - | - | -3.2x10^11^ |
|  | **Female AMH** | - | - | - | - | - |
| **t9**  **(n = 71)** | **Variable** | 98.2137 |  |  |  |  |
|  | **Male age** | 0.198 | 0.000 | - | - | -3.2x10^13^ |
|  | **Male BMI** | -0.476 | 0.000 | - | - | -5.7x10^11^ |
|  | **SDF** | 0.331 | 0.000 | - | - | -3.3x10^11^ |
|  | **Female age** | - | - | - | - | - |
|  | **Female BMI** | -0.577 | 0.000 | - | - | -7.3x10^8^ |
|  | **Female AMH** | -4.167 | 0.000 | - | - | -1.8x10^13^ |
| **tM**  **(n = 71)** | **Variable** | 71.8890 |  |  |  |  |
|  | **Male age** | 1.930 | 0.000 | - | - | -1.4x10^13^ |
|  | **Male BMI** | -1.342 | 0.000 | - | - | -4.6x10^11^ |
|  | **SDF** | 0.822 | 0.000 | - | - | -2.9x10^11^ |
|  | **Female age** | - | - | - | - | - |
|  | **Female BMI** | -1.8852 | 0.000 | - | - | -6.7x10^8^ |
|  | **Female AMH** | 1.220 | 0.000 | - | - | -1.8x10^13^ |
| **tB**  **(n = 62)** | **Variable** | 155.5643 |  |  |  |  |
|  | **Male age** | 1.333 | 0.000 | - | - | -3.2x10^13^ |
|  | **Male BMI** | -2.580 | 0.000 | - | - | -4.7x10^11^ |
|  | **SDF** | -0.415 | 0.000 | - | - | -2.6x10^11^ |
|  | **Female age** | - | - | - | - | - |
|  | **Female BMI** | -1.851 | 0.000 | - | - | -5.9x10^8^ |
|  | **Female AMH** | 0.804 | 0.000 | - | - | -1.4x10^13^ |
| **tEB**  **(n = 44)** | **Variable** | -9.607 |  |  |  |  |
|  | **Male age** | 1.873 | 0.000 | - | - | -4.0x10^13^ |
|  | **Male BMI** | 0.350 | 0.000 | - | - | -1.0x10^13^ |
|  | **SDF** | 0.478 | 0.000 | - | - | -8.9x10^12^ |
|  | **Female age** | 0.617 | 0.000 | - | - | -3.2x10^10^ |
|  | **Female BMI** | -0.306 | 0.000 | - | - | -2.1x10^13^ |
|  | **Female AMH** | 4.059 | 0.000 | - | - | -6.7x10^13^ |

**Abbreviations.** AMH, Anti-Müllerian hormone; BMI, Body mass index; SDF, Sperm DNA fragmentation; tPNa, time of pronuclei appearance; tPNf, time of pronuclei fading; tM,, Time of morula; tB, When the frame showed a crescent-shaped area began to emerge from the morula; tEB, Time when an increase in volume and expansion of the blastocoel cavity was visible.
